# Supplementary material for: FGCaMP7, an Improved Version of Fungi-Based Ratiometric Calcium Indicator for In Vivo Visualization of Neuronal Activity
Source: Int J Mol Sci. 2020 Apr 24;21(8):3012. doi: 10.3390/ijms21083012 (PMC7215472; doi:10.3390/ijms21083012)
Supplement: Supplementary file 1 [file ijms-21-03012-s001.zip › SupplementaryInformation/ijms-FGCaMP7 Supplementary Information_minor revision.docx]

**Supplementary Information**

FGCaMP7, an improved version of fungi-based ratiometric calcium indicator for in vivo visualization of neuronal activity

**Natalia V. Barykina, Vladimir P. Sotskov, Anna M. Gruzdeva, You Kure Wu, Ruben Portugues, Oksana M. Subach, Elizaveta S. Chefanova, Viktor V. Plusnin, Olga I. Ivashkina, Konstantin V. Anokhin, Anna V. Vlaskina, Dmitry A. Korzhenevskiy, Alena Y. Nikolaeva, Konstantin M. Boyko, Tatiana V. Rakitina, Anna M. Varizhuk, Galina E. Pozmogova, and Fedor V. Subach**

**Supplementary Figures and Tables**

**Table S1 2**

**Table S2 3**

**Table S3 4**

**Table S4 5**

**Table S5 6**

**Table S6 7**

**Table S7 8**

**Figure S1 9**

**Figure S2 10**

**Figure S3 11**

**Figure S4 12,13**

**Figure S5 14**

**Figure S6 15**

**Figure S7 16**

**Figure S8 17,18**

**Figure S9 19,20**

**Figure S10 21,22**

**Figure S11 23**

**Figure S12 24**

**Figure S13 25**

**Video S1 26**

**Video S2 27**

**Supplementary references 28**

**Table S1. Data collection, processing and refinement.**

| **Data collection** | |
| --- | --- |
| Diffraction source | BL41XU beamline, Spring8 |
| Wavelength (Å) | 1.0 |
| Temperature (K) | 100 |
| Detector | Pilatus |
| Crystal-to-detector distance (mm) | 570.00 |
| Rotation range per image (°) | 0.5 |
| Total rotation range (°) | 105 |
| Space group | P4_3_2_1_2 |
| *a*, *b*, *c* (Å) | 163.56; 163.56; 143.06 |
| α, β, γ (°) | 90.0 |
| Average mosaicity (°) | 0.45 |
| Unique reflections | 33,268 (4353) |
| Resolution range (Å) | 89.94–3.18  (3.34–3.18) |
| Completeness (%) | 100.0 (100.0) |
| Average redundancy | 7.0 (7.0) |
| 〈*I*/σ(*I*)〉 | 13.6 (2.4) |
| Rmeas (%) | 10.6 (94.7) |
| CC_1/2_ | 98.5 (72.6) |
| **Refinement** | |
| *R_fact_ (%)* | 27.9 |
| *R*_free._ *(%)* | 34.8 |
| Bonds (Å) | 0.01 |
| Angles (°) | 1.69 |
| *Ramachandran plot* |  |
| Most favored (%) | 94.5 |
| Allowed (%) | 3.6 |
| *No. atoms* |  |
| Protein | 6427 |
| Water | 3 |
| Chromophore | 44 |
| Calcium ion | 10 |
| *B-factors (Å^2^)* |  |
| Protein | 89.14 |
| Water | 45.19 |
| Chromophore | 77.44 |
| Calcium ion | 103.59 |

Values in parenthesis are for the highest-resolution shell.

**Table S2. In vitro contrasts of FGCaMP mutants**

| **Protein** | **Contrast for 402 nm-form** | **Contrast for 493 nm-form** |
| --- | --- | --- |
| **FGCaMP** | 6.9 ± 0.5 | 14.7 ± 0.6 |
| **FGCaMP/L21M** | 2.9 ± 0.3 | 17.6 ± 1.2 |
| **FGCaMP/N23L** | 1.6 ± 0.2 | 17.2 ± 7.8 |
| **FGCaMP/F39G*** | 1.0 ± 0.1 | 1.3 ± 0.1 |
| **FGCaMP/I41G** | 2.1 ± 0.3 | 3.3 ± 0.3 |
| **FGCaMP/V77G*** | 1.5 ± 0.2 | 1.5 ± 0.2 |
| **FGCaMP/S79A** | 0.30 ± 0.05 | 27.0 ± 1.4 |
| **FGCaMP/S79C** | 0.7 ± 0.1 | 6.2 ± 0.3 |
| **FGCaMP/V180G** | 2.2 ± 0.1 | 11.9 ± 0.4 |
| **FGCaMP/T181G*** | 0.9 ± 0.1 | 1.5 ± 0.2 |
| **FGCaMP/Q213A** | 3.9 ± 0.5 | 12.6 ± 2.7 |
| **FGCaMP/R215A*** | 0.8 ± 0.1 | 1.6 ± 0.2 |
| **FGCaMP/T265S** | 3.4 ± 0.4 | 14.1 ± 2.9 |
| **FGCaMP/L266G** | 1.9 ± 0.2 | 1.9 ± 0.3 |
| **FGCaMP/M267G** | 1.5 ± 0.1 | 10.7 ± 1.3 |

* Mutations that impaired FGCaMP folding.

**Table S3. Mutations in FGCaMP variants described in this work**

| **Protein** | **Mutations relative to FGCaMP^*^** |
| --- | --- |
| FGCaMP2 | N327D/D345Y/T346R/N364D |
| FGCaMP3 | N327D/D345Y/T346R/N364D/S368D |
| FGCaMP4 | T3L/H5K/N327D/D345Y/T346R/N364D/S368D |
| FGCaMP4.03 | T3L/H5K/L21M/D221G/N327D/K342E/D345Y/T346R/S348T/A355V/N364D/S368D |
| FGCaMP4.05 | T3L/H5K/L21M/K122R/F218L/D221G/D325G/N326S/N327D/K342E/D345Y/  T346R/S348T/A355V/N364D/S368D |
| FGCaMP5 | T3L/H5K/**N5I**/L21M/D47G/K122R/F218L/D221G/T265S/D325G/N326S/N327D/  K342E/D345Y/T346R/S348T/A355V/N364D/S368D |
| FGCaMP6 | T3L/H5K/L21M/D47G/K122R/**T162A**/F218L/D221G/T265S/**T272A**/D325G/  N326S/N327D/K342E/D345Y/T346R/S348T/A355V/N364D/S368D |
| FGCaMP7 | T3L/H5K/L21M/D47G/**K88Q**/K122R/F218L/D221G/T265S/D325G/N326S/N327D/K342E/D345Y/T346R/S348T/A355V/N364D/S368D |

^*^ Substitution number follows that of FGCaMP sequence. Unique mutations for the novel FGCaMP5, FGCaMP6, and FGCaMP7 variants are bold.

**Table S4. In vitro characterization of FGCaMP5, FGCaMP6, and FGCaMP7 indicators**

|  | | **Proteins** | | | | | | |
| --- | --- | --- | --- | --- | --- | --- | --- | --- |
| **Properties** | | **FGCaMP7** | | **FGCaMP5** | | **FGCaMP6** | | |
|  |  | **apo** | **sat** | **apo** | **sat** | **apo** | **sat** | |
| **Absorbance maximum (nm)** | | 400 | 498 | 400 | 497 | 400 | 496 | |
| **Emission maximum (nm)** | | 516 | | 516 | | 516 | | |
| **Quantum yield ^a^** | | 0.4 ± 0.02 | 0.55 ± 0.04 | 0.57 ± 0.04 | 0.56 ± 0.05 | 0.32 ± 0.01 | 0.53 ± 0.04 | |
| **ε (mM^-1^cm^-1^) ^b^** | | 82 ± 11 | 103 ± 14 | 77 ± 13 | 119 ± 16 | 54 ± 10 | 104 ± 11 | |
| **Brightness (%) ^c^** | | 123 | 116 | 166 | 137 | 65 | 113 | |
| **Fluorescence contrast (fold) with 1 mM MgCl_2_** | **Ex 400** | 10.1 ± 1.0 | | 8.0 ± 0.4 | | 9.0 ± 0.1 | | |
|  | **Ex 498** | 32.7 ± 1.5 | | 17.6 ± 1.1 | | 33.0 ± 0.3 | | |
| **p*K*a ^d^** | **Ex 400** | 6.63 ± 0.20 | 6.00 ± 0.20 | 7.01 ± 0.03 | 5.75 ± 0.15 | 6.84 ± 0.02 | | 5.57 ± 0.04 |
|  | **Ex 498** | 5.28 ± 0.10  7.81 ± 0.10 | 6.87 ± 0.01 | 6.44 ± 0.09 | 6.86 ± 0.03 | 6.80 ± 0.01 | | 6.88 ± 0.03 |
| **K_d_ (nM) with 1 mM MgCl_2_ ^e^** | **Ex 400** | 230 ± 5 (n = 2.2 ± 0.1) | | 87 ± 1 (n = 1.6 ± 0.1) | | 240 ± 60 (n = 1.8 ± 0.1) | | |
|  | **Ex 498** | 240 ± 6 (n = 2.3 ± 0.2) | | 98 ± 3 (n = 1.8 ± 0.2) | | 350 ± 13 (n = 1.8 ± 0.1) | | |
| **k_obs_ (s^-1^)**  **(300 nM Ca^2+^) ^f^** | **Ex 400** | 0.60 ± 0.01 | | 6.3 ± 0.4; 1.01 ± 0.01 | | 5.2 ± 0.1; 0.80 ± 0.01 | | |
|  | **Ex 498** | 0.42 ± 0.01 | | 0.81 ± 0.01 | | 0.52 ± 0.02 | | |
| **t_1/2_^off^ (s) ^g^** | **Ex 400** | 1.5 ± 0.1 | | 2.2 ± 0.1 | | 1.2 ± 0.1 | | |
|  | **Ex 498** | 1.34 ± 0.02 | | 2.01 ± 0.02 | | 0.97 ± 0.02 | | |

^a^ mEGFP (QY = 0.61[[1](#_ENREF_1)]) and mTagBFP2 (QY = 0.64 [[2](#_ENREF_2)]) were used as reference standards for 496–498 and 400 nm absorbing states, respectively.

^b^ Extinction coefficient was determined by alkaline denaturation.

^c^ Brightness normalized to brightness of FGCaMP indicator.

^d^ p*K*a values were determined according to pH dependence of fluorescence.

^e^ Experimental data was fitted to Hill equation. Hill coefficients are shown in square brackets. K_d_ for GCaMP6s 144 ± 9 nM (4.0 ± 0.6); in the presence of 1 mM MgCl_2_ K_d_ for GCaMP6s 217 ± 16 nM (4.0 ± 0.6).

^f^ Observed Ca^2+^-associated rate constants were determined from associated curves in the presence of 1 mM MgCl_2_. k_obs_ for GCaMP6s is 0.49 ± 0.01 s^-1^, k_obs_ for GCaMP6f is 1.28 ± 0.01 s^-1^.

^g^ t_1/2_^off^ values were determined from the dissociation kinetics curves in the presence of 1 mM MgCl_2_. t_1/2_^off^ for GCaMP6s is 1.01 ± 0.06 s; t_1/2_^off^ for GCaMP6f is 0.37 ± 0.04 s.

**Table S5.** **In vitro ΔF/F_0_ response of truncated versions (with deleted M13-like peptide) of the purified FGCaM7 and GCaM6s indicators to the saturating calcium ion concentrations.**

| **Indicator** | **ΔF/F_0_, %** | | |
| --- | --- | --- | --- |
|  | **39 µM ^a^** | **820 µM ^a^** | **2000 µM ^a^** |
| **FGCaM7** | -4 ± 10 | -13 ± 6 | -16 ± 2 |
| **GCaM6s** | 5 ± 18 | -9 ± 18 | 10 ± 12 |

^a^ 39, 820, and 2000 µM free calcium concentration corresponds to the buffer supplemented with 10 mM CaEGTA, 10 mM NTA or 2 mM CaCl_2_, respectively. Data were averaged across 3–6 repeats. SD is shown.

**Table S6.** **Comparison of calcium responses in neurons expressing FGCaMP7 and GCaMP6s indicators in hippocampus of freely moving mice registered with nVista HD miniscope in vivo.**

| **Properties** | **Indicators** | |
| --- | --- | --- |
|  | FGCaMP7 | GCaMP6s |
| **Number of mice** | 2 | 4 |
| **Number of active cells ^a^** | 142 | 436 |
| **Number of spikes detected** | 135 | 316 |
| **Rise half-time, s ^b^** | 0.97 ± 0.60 (*p* = 0.0002) | 0.76 ± 0.49 |
| **Decay half-time, s ^b^** | 3.07 ± 0.80 (*p* < 0.0001) | 2.54 ± 0.94 |
| **Peak ΔF/F_0_ ^c^** | 1.0 ± 1.2 (*p* < 0.0001) | 2.1 ± 1.8 |

^a^ Only spikes exceeding 4MAD threshold and not less than 50% of maximum value of the trace were taken into account.

^b^ Rise and decay half-times were calculated as time intervals between the peak of the mean spike and half-peak at front and back slopes of the spike.

^c^ Peak ΔF/F_0_ is given in MIN1PIPE units, which are ΔF/F_0_ units after normalization and registration.

^b-c^ Mean values ± standard error of mean are given. Analysis of calcium imaging data was performed as previously described [[3](#_ENREF_3)].

*p* value shows statistical difference between the respective values for NCaMP7 and GCaMP6s control indicator.

**Table S7. List of primers.**

| **Primer** | **Primer sequence (5’-3’)** |
| --- | --- |
| **FGCaMP-L21M** | CAACAAGCTCCGCGAGGGAATGGGCAACGTCTATATCAAG |
| **FGCaMP-L21M-r** | CTTGATATAGACGTTGCCCATTCCCTCGCGGAGCTTGTTG |
| **FGCaMP-N23L** | CTCCGCGAGGGATTGGGCCTGGTCTATATCAAGGCCGAC |
| **FGCaMP-N23L-r** | GTCGGCCTTGATATAGACCAGGCCCAATCCCTCGCGGAG |
| **FGCaMP-F39G** | GAACGGCATCAAGGCGAACGGAAAGATCCGCCACAACATC |
| **FGCaMP-F39G-r** | GATGTTGTGGCGGATCTTTCCGTTCGCCTTGATGCCGTTC |
| **FGCaMP-I41G** | CATCAAGGCGAACTTCAAGGGACGCCACAACATCGAGGAC |
| **FGCaMP-I41G-r** | GTCCTCGATGTTGTGGCGTCCCTTGAAGTTCGCCTTGATG |
| **FGCaMP-V77G** | GACAACCACTACCTGAGCGGTCAGTCCAAACTTTCGAAAG |
| **FGCaMP-V77G-r** | CTTTCGAAAGTTTGGACTGACCGCTCAGGTAGTGGTTGTC |
| **FGCaMP-S79A** | CACTACCTGAGCGTGCAGGCCAAACTTTCGAAAGACCCC |
| **FGCaMP-S79A-r** | GGGGTCTTTCGAAAGTTTGGCCTGCACGCTCAGGTAGTG |
| **FGCaMP-S79C** | CACTACCTGAGCGTGCAGTGCAAACTTTCGAAAGACCCC |
| **FGCaMP-S79C-r** | GGGGTCTTTCGAAAGTTTGCACTGCACGCTCAGGTAGTG |
| **FGCaMP-V180G** | GTGCCCTGGCCCACCCTCGGAACCACCCTGACCTACGGC |
| **FGCaMP-V180G-r** | GCCGTAGGTCAGGGTGGTTCCGAGGGTGGGCCAGGGCAC |
| **FGCaMP-T181G** | CCCTGGCCCACCCTCGTGGGAACCCTGACCTACGGCGTG |
| **FGCaMP-T181G-r** | CACGCCGTAGGTCAGGGTTCCCACGAGGGTGGGCCAGGG |
| **FGCaMP-Q213A** | CATGCCCGAAGGCTACATCGCTGAGCGCACCATCTTCTTC |
| **FGCaMP-Q213A-r** | GAAGAAGATGGTGCGCTCAGCGATGTAGCCTTCGGGCATG |
| **FGCaMP-R215A** | GAAGGCTACATCCAGGAGGCTACCATCTTCTTCAAGGAC |
| **FGCaMP-R215A-r** | GTCCTTGAAGAAGATGGTAGCCTCCTGGATGTAGCCTTC |
| **FGCaMP-T265S** | CAAGCTGGAGTACGACGTCAGCCTGATGGCCGACTCTCTG |
| **FGCaMP-T265S-r** | CAGAGAGTCGGCCATCAGGCTGACGTCGTACTCCAGCTTG |
| **FGCaMP-L266G** | CTGGAGTACGACGTCACCGGCATGGCCGACTCTCTGACC |
| **FGCaMP-L266G-r** | GGTCAGAGAGTCGGCCATGCCGGTGACGTCGTACTCCAG |
| **FGCaMP-M267G** | GAGTACGACGTCACCCTGGGAGCCGACTCTCTGACCGAAG |
| **FGCaMP-M267G-r** | CTTCGGTCAGAGAGTCGGCTCCCAGGGTGACGTCGTACTC |
| **TorA** | CGAccatgggtttaaagaggagaaag |
| **CaMN-HindIII-r** | CCAAGCTTTTATTTTTGCATCATGAGCTGGACGAACTCGTTG |


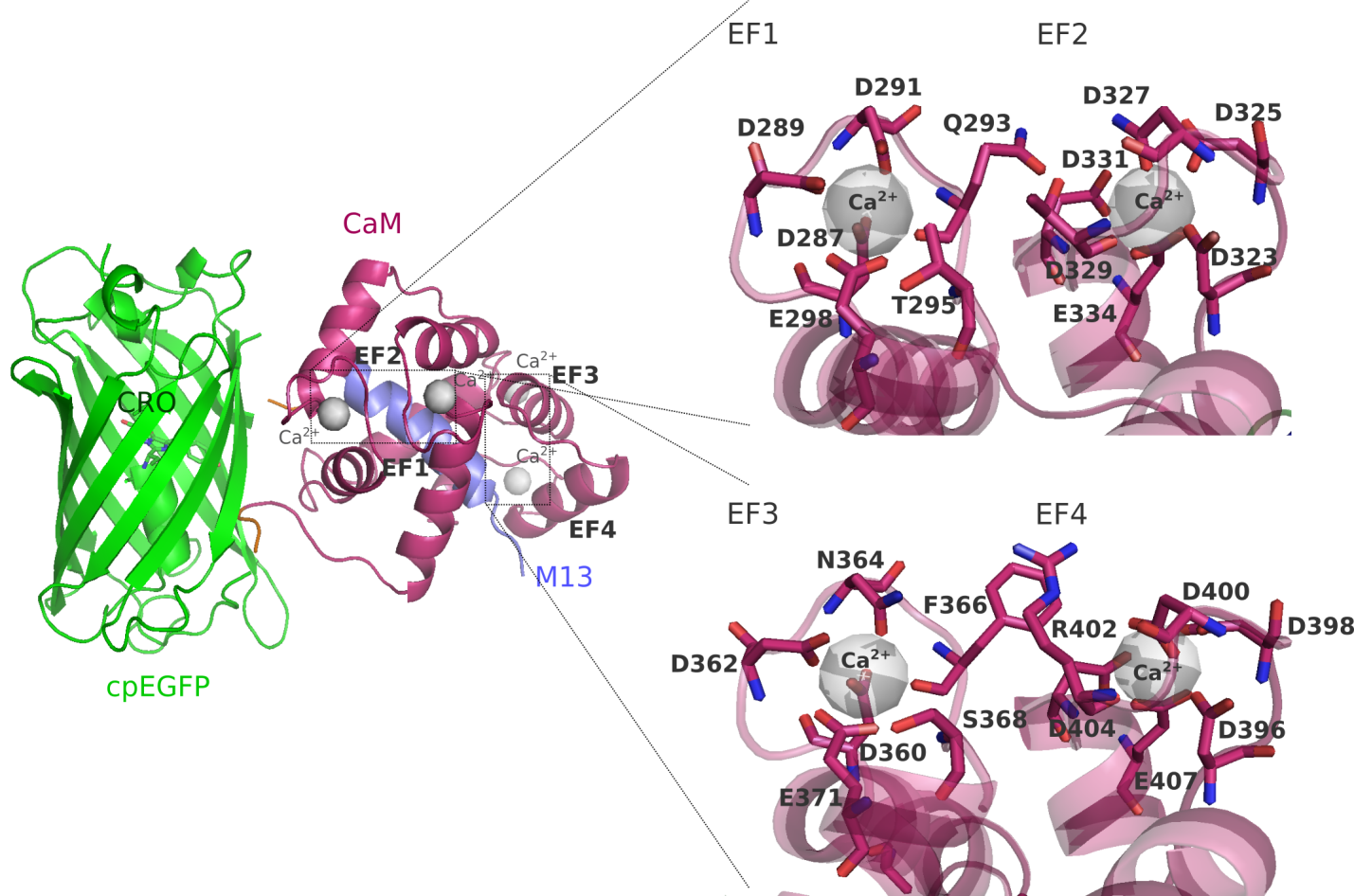


**Figure S1. Crystal structure of FGCaMP indicator in Ca^2+^-bound state (PDB 6XU4). (Left)** β-barrel of cpEGFP is shown as a green cylinder, Ca^2+^ ions are shown as grey spheres, M13-peptide and CaM are shown in purple and light blue, respectively. Cro: cromophore. **(Right)** Ca^2+^-binding motives 1 and 2, 3 and 4 (EF1–EF4). Ca^2+^ ions are shown as grey spheres. Ca^2+^-binding amino acids are indicated.


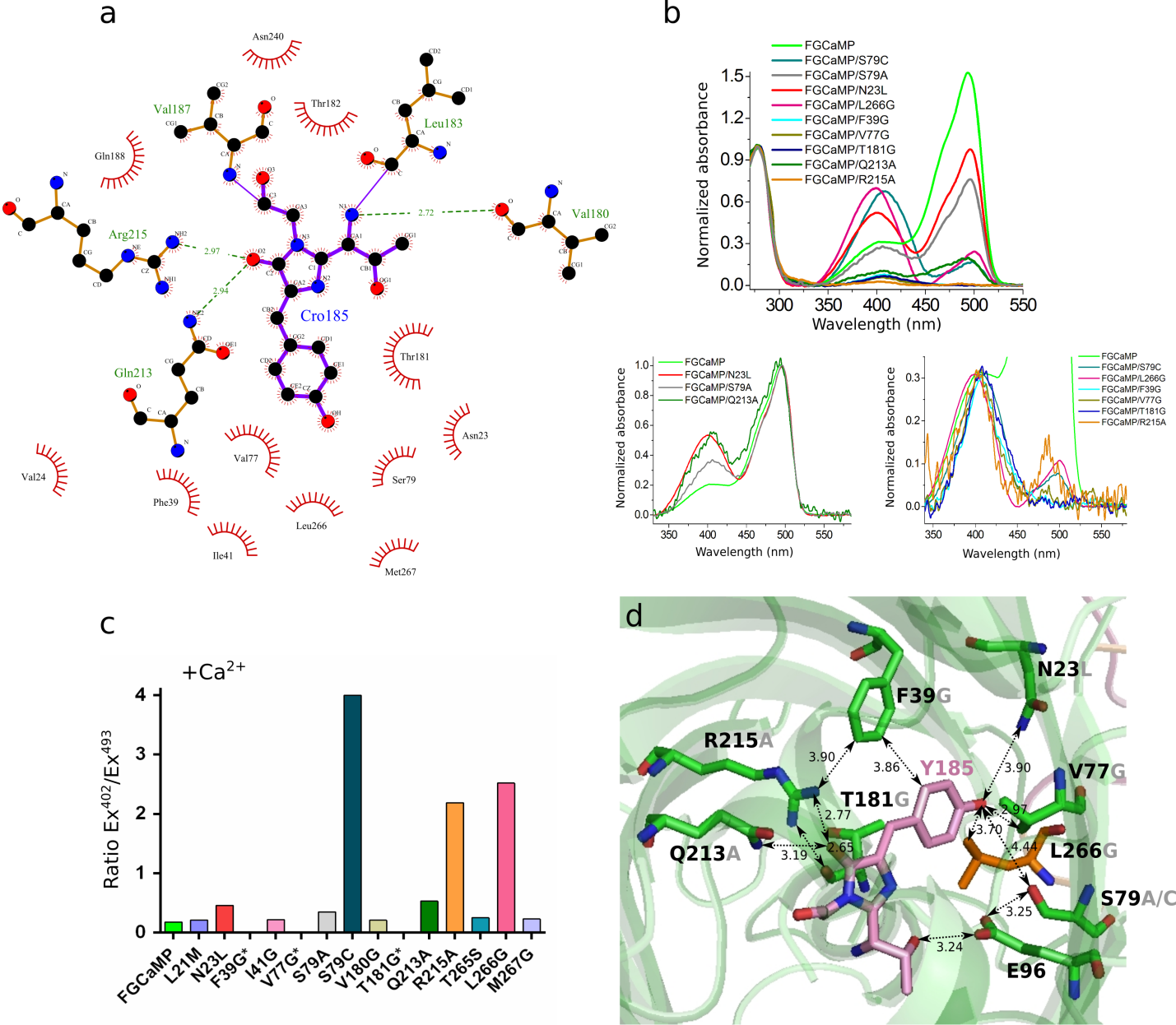


**Figure S2. Absorbance ratio for two forms of calcium indicator FGCaMP and its mutants. (a)** The immediate surroundings of the chromophore. **(b, top)** Absorbance for FGCaMP and its nine mutants in Ca^2+^-saturated state. Absorbance spectra are presented only for nine FGCaMP mutants with substantial change in the Abs^402^/Abs^493^ ratio. Absorbance at 280 nm is normalized to 1. **(b, bottom left)** Absorbance spectra of FGCaMP mutants with dominant 493 nm absorbing form. Absorbance at 493 nm is normalized to 1. **(b, bottom right)** Absorbance spectra of FGCaMP mutants with dominant 402-nm absorbing form. Absorbance at 402 nm is normalized to 0.3. **(c)** Abs^402^/Abs^493^ ratios are presented for all 14 FGCaMP mutants in 13 amino acid positions in Ca^2+^-saturated state. Mutants with absent anionic 493 nm absorbing form are marked with asterisk. **(d)** Chromophore and eight nearby mutated amino acid residues of the FGCaMP indicator (PDB 6XU4). Only residues leading to substantial change in the Abs^402^/Abs^493^ ratio are shown. Distances between mutated amino acids and chromophore are indicated above black arrows. Amino acid codes and numberings in initial FGCaMP are in black; mutation codes are in light grey. Chromophore residue, residues from fluorescent domain and linker are shown in pink, green and orange, respectively.


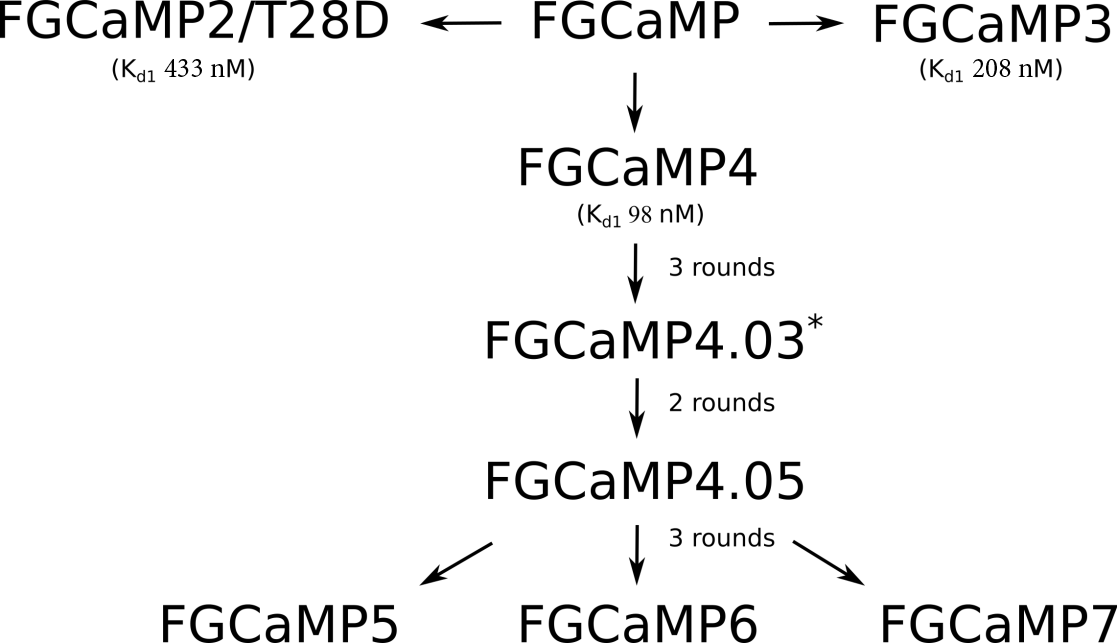


**Figure S3. Genealogy of FGCaMP variants.**

* Beginning from the third round mutants have monophasic calcium-binding curve with single dissociation constant (K_d_).

10 20 30 40 50 60

| | | | | |

FGCaMP RRTLHKAIDTVRAINKLREGLGNVYIKADKQKNGIKANFKIRHNIEDGGVQLAYHYQQNT

FGCaMP7 RRLLKKAIDTVRAINKLREGMGNVYIKADKQKNGIKANFKIRHNIEGGGVQLAYHYQQNT

FGCaMP5 RRLLKKAIDTVRAIIKLREGMGNVYIKADKQKNGIKANFKIRHNIEGGGVQLAYHYQQNT

FGCaMP6 RRLLKKAIDTVRAINKLREGMGNVYIKADKQKNGIKANFKIRHNIEGGGVQLAYHYQQNT

GCaMP6f RRKWNKTGHAVRAIGRLSS-LENVYIKADKQKNGIKANFKIRHNIEDGGVQLAYHYQQNT

70 80 90 100 110 120

| | | | | |

FGCaMP PIGDGPVLLPDNHYLSVQSKLSKDPNEKRDHMVLLEFVTAAGITLGMDELYKGGTGGSMV

FGCaMP7 PIGDGPVLLPDNHYLSVQSKLSKDPNEQRDHMVLLEFVTAAGITLGMDELYKGGTGGSMV

FGCaMP5 PIGDGPVLLPDNHYLSVQSKLSKDPNEKRDHMVLLEFVTAAGITLGMDELYKGGTGGSMV

FGCaMP6 PIGDGPVLLPDNHYLSVQSKLSKDPNEKRDHMVLLEFVTAAGITLGMDELYKGGTGGSMV

GCaMP6f PIGDGPVLLPDNHYLSVQSKLSKDPNEKRDHMVLLEFVTAAGITLGMDELYKGGTGGSMV

130 140 150 160 170 180

| | | | | |

FGCaMP SKGEELFTGVVPILVELDGDVNGHKFSVSGEGEGDATYGKLTLKFICTTGKLPVPWPTLV

FGCaMP7 SRGEELFTGVVPILVELDGDVNGHKFSVSGEGEGDATYGKLTLKFICTTGKLPVPWPTLV

FGCaMP5 SRGEELFTGVVPILVELDGDVNGHKFSVSGEGEGDATYGKLTLKFICTTGKLPVPWPTLV

FGCaMP6 SRGEELFTGVVPILVELDGDVNGHKFSVSGEGEGDATYGKLALKFICTTGKLPVPWPTLV

GCaMP6f SKGEELFTGVVPILVELDGDVNGHKFSVSGEGEGDATYGKLTLKFICTTGKLPVPWPTLV

190 200 210 220 230 240

*** | | | | | |

FGCaMP TTLTYGVQCFSRYPDHMKQHDFFKSAMPEGYIQERTIFFKDDGYYKTRAEVKFEGDTLVN

FGCaMP7 TTLTYGVQCFSRYPDHMKQHDFFKSAMPEGYIQERTILFKGDGYYKTRAEVKFEGDTLVN

FGCaMP5 TTLTYGVQCFSRYPDHMKQHDFFKSAMPEGYIQERTILFKGDGYYKTRAEVKFEGDTLVN

FGCaMP6 TTLTYGVQCFSRYPDHMKQHDFFKSAMPEGYIQERTILFKGDGYYKTRAEVKFEGDTLVN

GCaMP6f TTLTYGVQCFSRYPDHMKQHDFFKSAMPEGYIQERTIFFKDDGNYKTRAEVKFEGDTLVN

250 260 270 280 290 300

| | | | | |

FGCaMP RIELKGIDFKEDGNILGHKLEYDVTLMADSLTEEQVSEYKEAFSLFDKDGDGQITTKELG

FGCaMP7 RIELKGIDFKEDGNILGHKLEYDVSLMADSLTEEQVSEYKEAFSLFDKDGDGQITTKELG

FGCaMP5 RIELKGIDFKEDGNILGHKLEYDVSLMADSLTEEQVSEYKEAFSLFDKDGDGQITTKELG

FGCaMP6 RIELKGIDFKEDGNILGHKLEYDVSLMADSLAEEQVSEYKEAFSLFDKDGDGQITTKELG

GCaMP6f RIELKGIDFKEDGNILGHKLEYNLP---DQLTEEQIAEFKEEFSLFDKDGDGTITTKELG

310 320 330 340 350 360

| | | | | |

FGCaMP TVMRSLDQNPSESELQDMINEVDADNNGTIDFPEFLTMMARKMKDTDSEEEIREACKVFD

FGCaMP7 TVMRSLDQNPSESELQDMINEVDAGSDGTIDFPEFLTMMAREMKYRDTEEEIREVCKVFD

FGCaMP5 TVMRSLDQNPSESELQDMINEVDAGSDGTIDFPEFLTMMAREMKYRDTEEEIREVCKVFD

FGCaMP6 TVMRSLDQNPSESELQDMINEVDAGSDGTIDFPEFLTMMAREMKYRDTEEEIREVCKVFD

GCaMP6f TVMRSLGQNPTEAELQDMINEVDADGDGTIDFPEFLTMMARKMKYRDTEEEIREAFGVFD

370 380 390 400 410

| | | | |

FGCaMP RDNNGFISAAELRHVMTSIGEKLTDDEVDEMIREADQDGDGRIDYNEFVQLMMQK

FGCaMP7 RDNDGFIDAAELRHVMTSIGEKLTDDEVDEMIREADQDGDGRIDYNEFVQLMMQK

FGCaMP5 RDNDGFIDAAELRHVMTSIGEKLTDDEVDEMIREADQDGDGRIDYNEFVQLMMQK

FGCaMP6 RDNDGFIDAAELRHVMTSIGEKLTDDEVDEMIREADQDGDGRIDYNEFVQLMMQK

GCaMP6f KDGNGYISAAELRHVMTNLGEKLTDEEVDEMIREADIDGDGQVNYEEFVQMMTAK

**Figure S4. Alignment of the amino acid sequences for FGCaMP, FGCaMP5, FGCaMP6, FGCaMP7, and GCaMP6f calcium indicators.** Alignment number follows that of FGCaMP. Residues of fluorescent part buried in β-barrel are highlighted with green. Residues that are forming chromophore are marked with asterisks. Calcium-binding EF1–EF4 hands are highlighted with cyan. Linkers between fluorescent and calcium-binding parts are highlighted with yellow. Mutations in FGCaMP variants relative to the original FGCaMP are highlighted with red. Secondary structure of FGCaMP is presented on top.


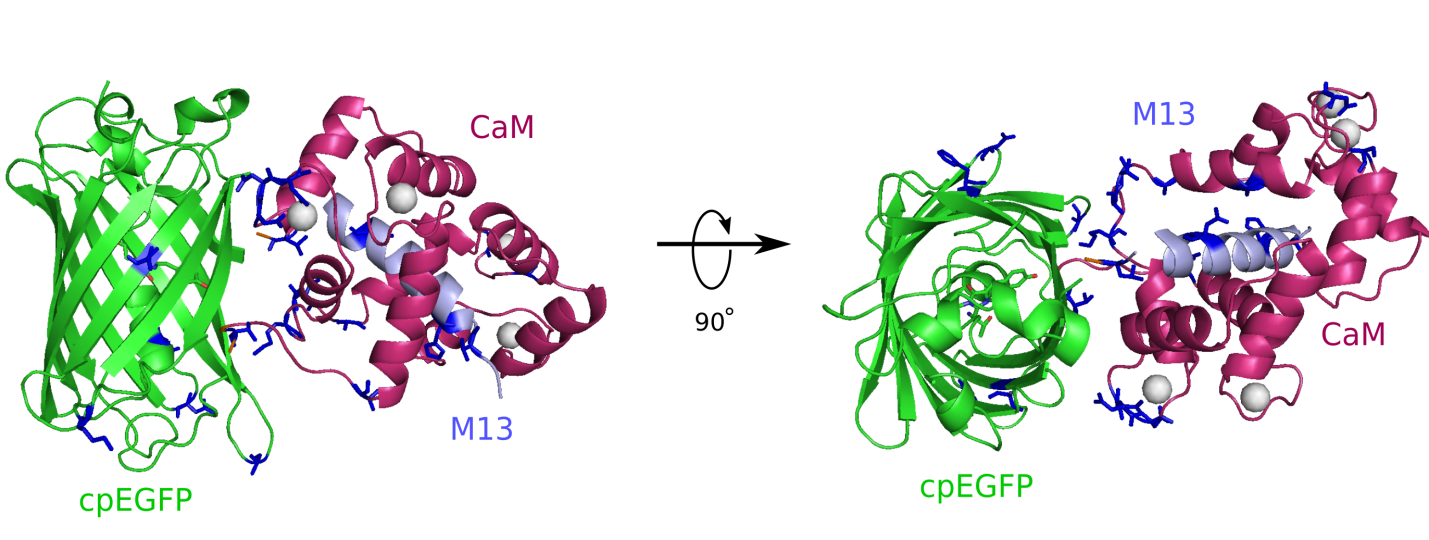


**Figure S5. Cartoon representation of FGCaMP calcium indicator with mutations from FGCaMP5, FGCaMP6, and FGCaMP7 variants.** Orthogonal views of the structure of FGCaMP indicator in Ca^2+^-bound state (PDB 6XU4). β-barrel of cpEGFP is shown as a green cylinder, Ca^2+^ ions are shown as grey spheres, M13-peptide and CaM are shown in purple and light blue, respectively. Positions of FGCaMP5, FGCaMP6, and FGCaMP7 mutations relative to FGCaMP structure are depicted as blue sticks.


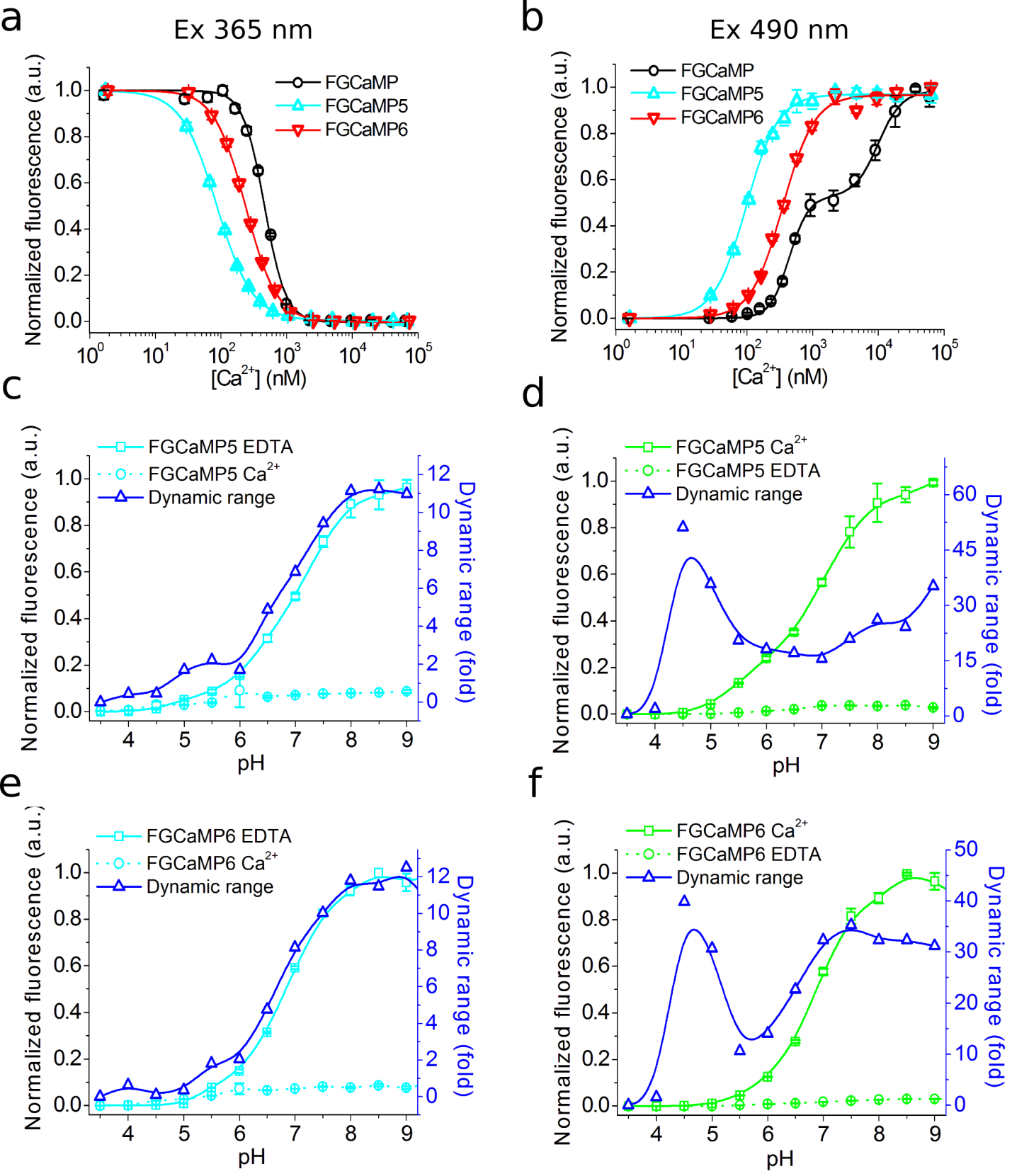


**Figure S6. Calcium titration curves and pH dependences for FGCaMP5 and FGCaMP6. (a)** Ca^2+^ titration curves for FGCaMP5 and FGCaMP6 at 365 nm excitation. **(b)** Ca^2+^ titration curves for FGCaMP5 and FGCaMP6 at 490 nm excitation. **(c)** Fluorescence of FGCaMP5 as a function of pH at 365 nm excitation. **(d)** Fluorescence of FGCaMP5 as a function of pH at 490 nm excitation. **(e)** Fluorescence of FGCaMP6 as a function of pH at 365 nm excitation. **(f)** Fluorescence of FGCaMP6 as a function of pH at 490 nm excitation.


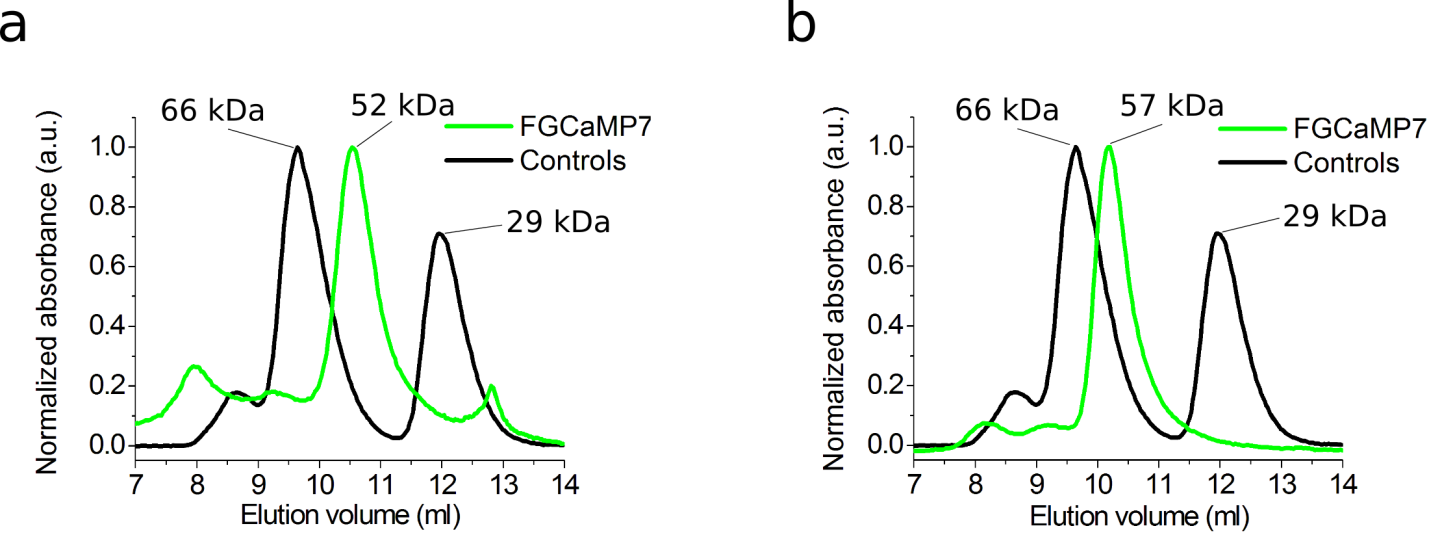


**Figure S7. Size-exclusion chromatography for FGCaMP7 protein.** **(a)** Fast protein liquid chromatography of FGCaMP7 in 20 mM Tris-HCl (pH 7.5), 100 mM NaCl buffer supplemented with 1 mM CaCl_2_ or **(b)** in 20 mM Tris-HCl (pH 7.5), 200 mM NaCl buffer supplemented with 2 mM EDTA.


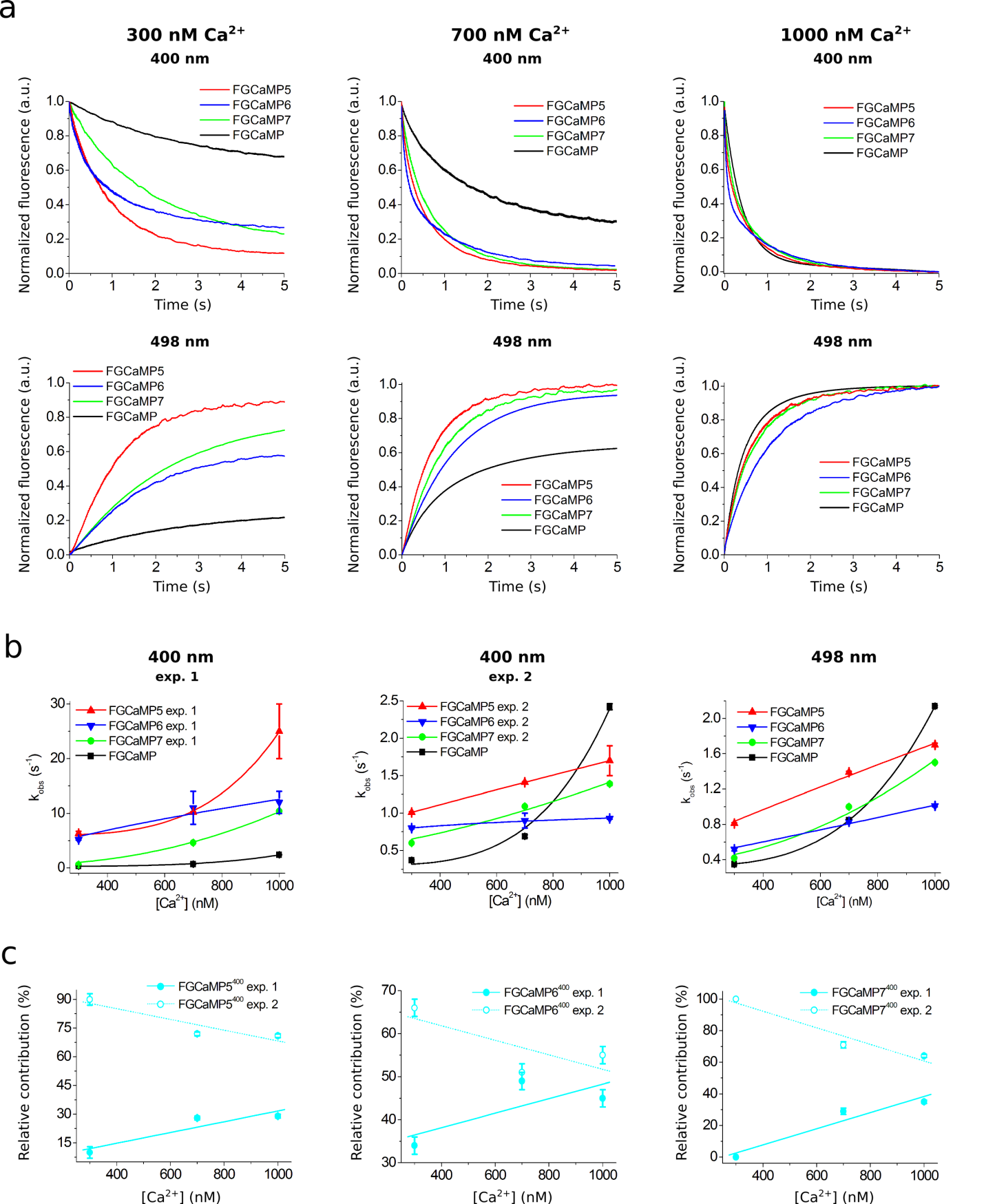


**Figure S8.** **Calcium-association kinetics for the FGCaMP indicator series characterized by stopped-flow fluorimetry.** **(a)** Calcium-association kinetics curves for FGCaMP, FGCaMP5, FGCaMP6, and FGCaMP7 GECIs at Ca^2+^ concentrations of 300 nM, 700 nM, and 1000 nM. Calcium-association curves are shown for both forms with maximum excitation at 400 and 498 nm. **(b)** Observed Ca^2+^-association rate constants determined from association curves for FGCaMP, FGCaMP5, FGCaMP6, and FGCaMP7 GECIs. For the form with excitation maximum at 400 nm fast (exp. 1) and slow (exp. 2) exponents are shown. The data was fitted to the equation k_obs_ = k_on_ × [Ca^2+^]^n^ + k_off_. **(c)** Relative contribution of monoexponents A1/(A1 + A2) and A2/(A1 + A2) for FGCaMP5, FGCaMP6, and FGCaMP7 GECIs at 400 nm excitation, where A1 and A2 are the pre-exponential factors in the association curve equation ΔFlu(t) = A1 × exp(-k^on^_obs1_ × t) – A2 × exp(-k^on^_obs2_ × t).


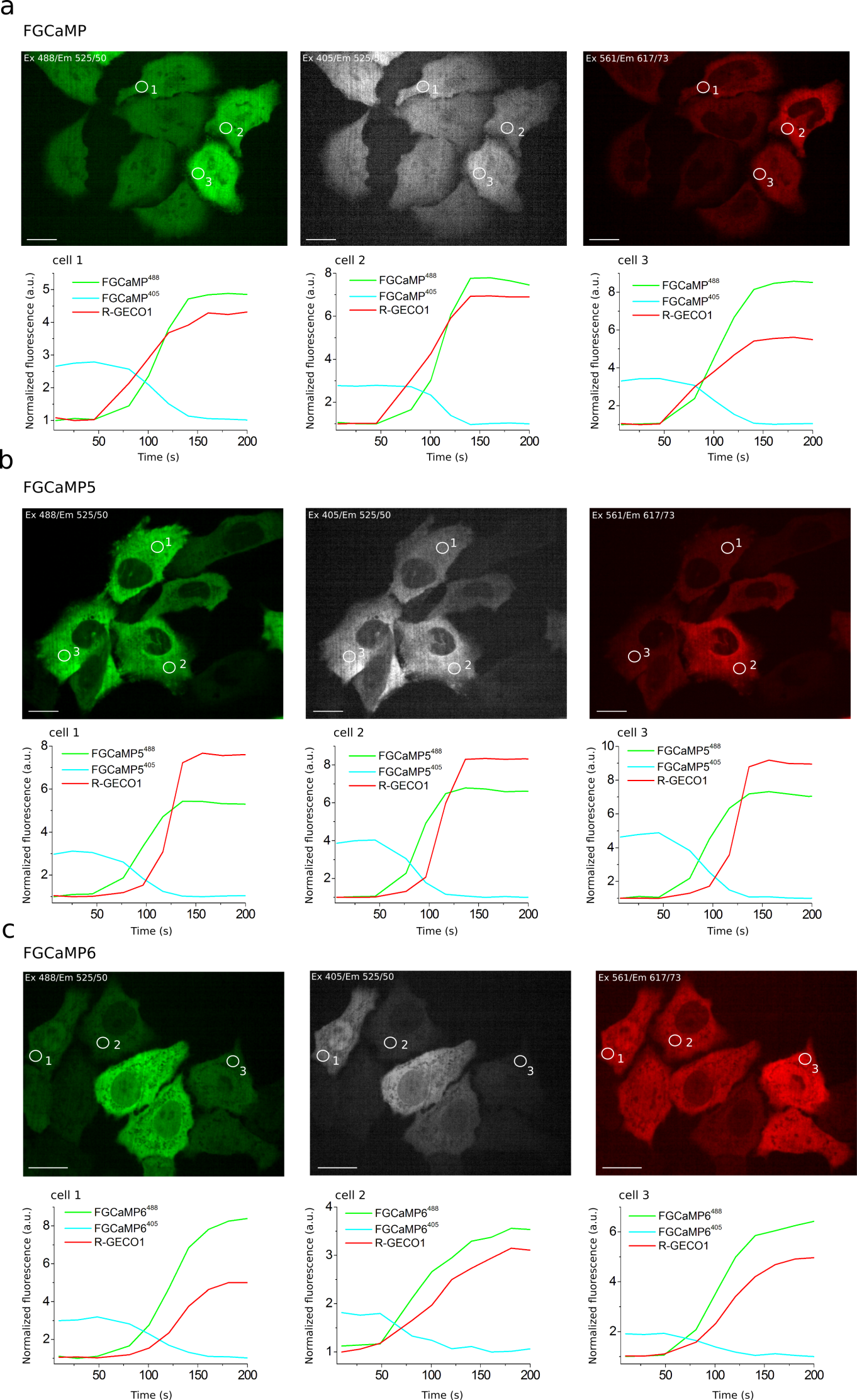


**Figure S9. Response of FGCaMP (a), FGCaMP5 (b), and FGCaMP6 (c) GECIs to variations in the cytoplasmic calcium concentration in HeLa Kyoto cells.** **(Upper panels)** Confocal images of HeLa Kyoto cells co-expressing respective indicator from FGCaMP series at 488 nm (left panels) and 405 nm (middle panels) excitations and red indicator R-GECO1 at 561 nm excitation (right panels). **(Lower panels)** The graphs illustrate changes in green fluorescence of the respective indicator from FGCaMP series at 488 nm (green lines) and 405 nm (cyan lines) excitations and red fluorescence of co-expressed R-GECO1 (red lines) GECI in response to 2.5 μM ionomycin. Changes in fluorescence of each FGCaMP indicator are shown for three cells. The changes shown in graphs correspond to the areas indicated with white circles in the images in upper panels. Scale bars: 20 μm.


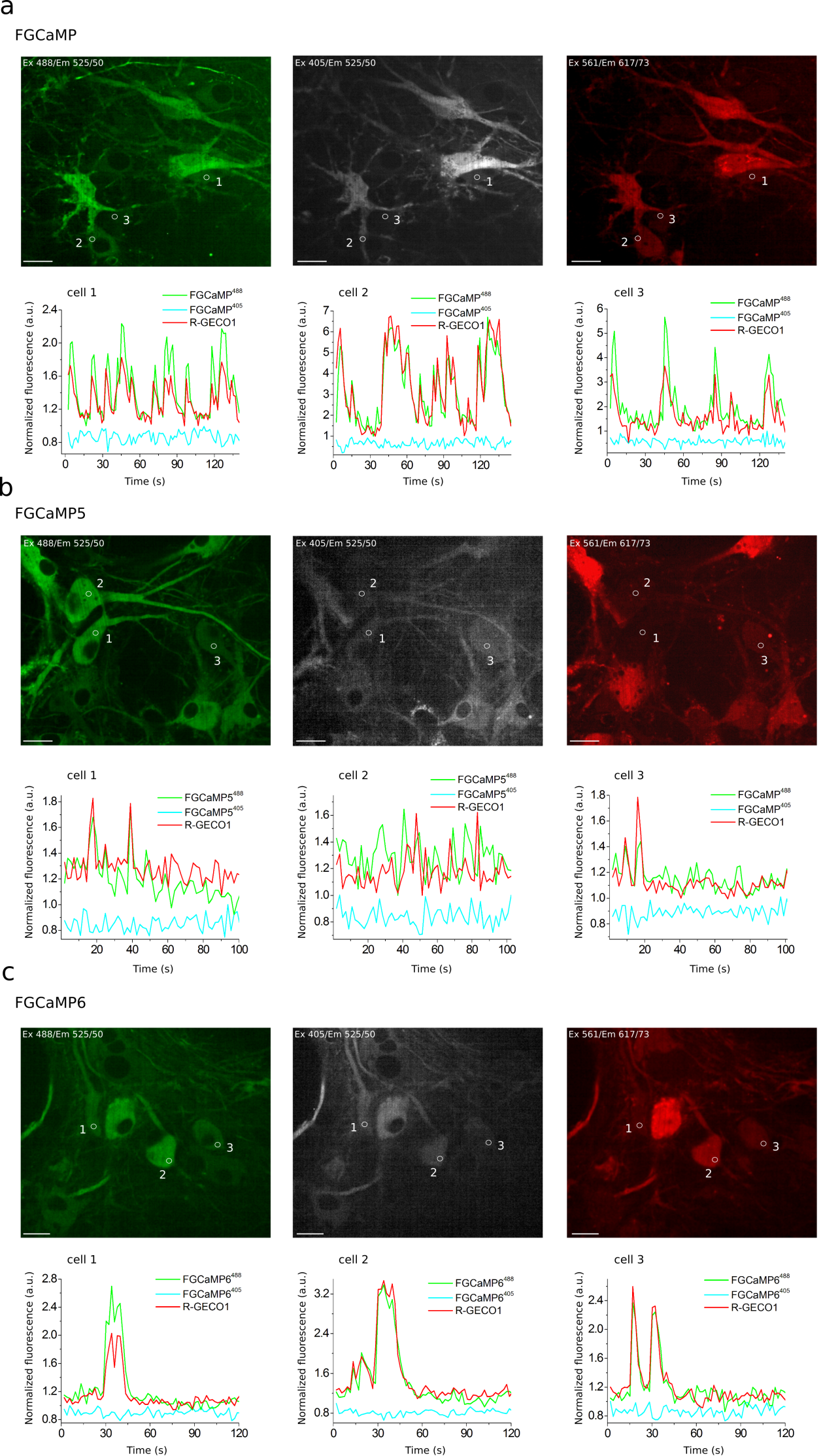


**Figure S10. Response of FGCaMP (a), FGCaMP5 (b) and FGCaMP6 (c) GECIs to variations in the cytoplasmic calcium concentration in cultured neurons.** **(Upper panels)** Confocal images of neurons co-expressing respective indicator from FGCaMP series at 488 nm (left panels) and 405 nm (middle panels) excitations and red indicator R-GECO1 at 561 nm excitation (right panels). **(Lower panels)** The graphs illustrate changes in green fluorescence of the respective indicator from FGCaMP series at 488 nm (green lines) and 405 nm (cyan lines) excitations and red fluorescence of co-expressed R-GECO1 (red lines) GECI as a result of spontaneous activity in neuronal cultures. Changes in fluorescence of each FGCaMP indicator are shown for three neurons. The changes shown in graphs correspond to the areas indicated with white circles in the images in upper panels. Scale bars: 20 μm.


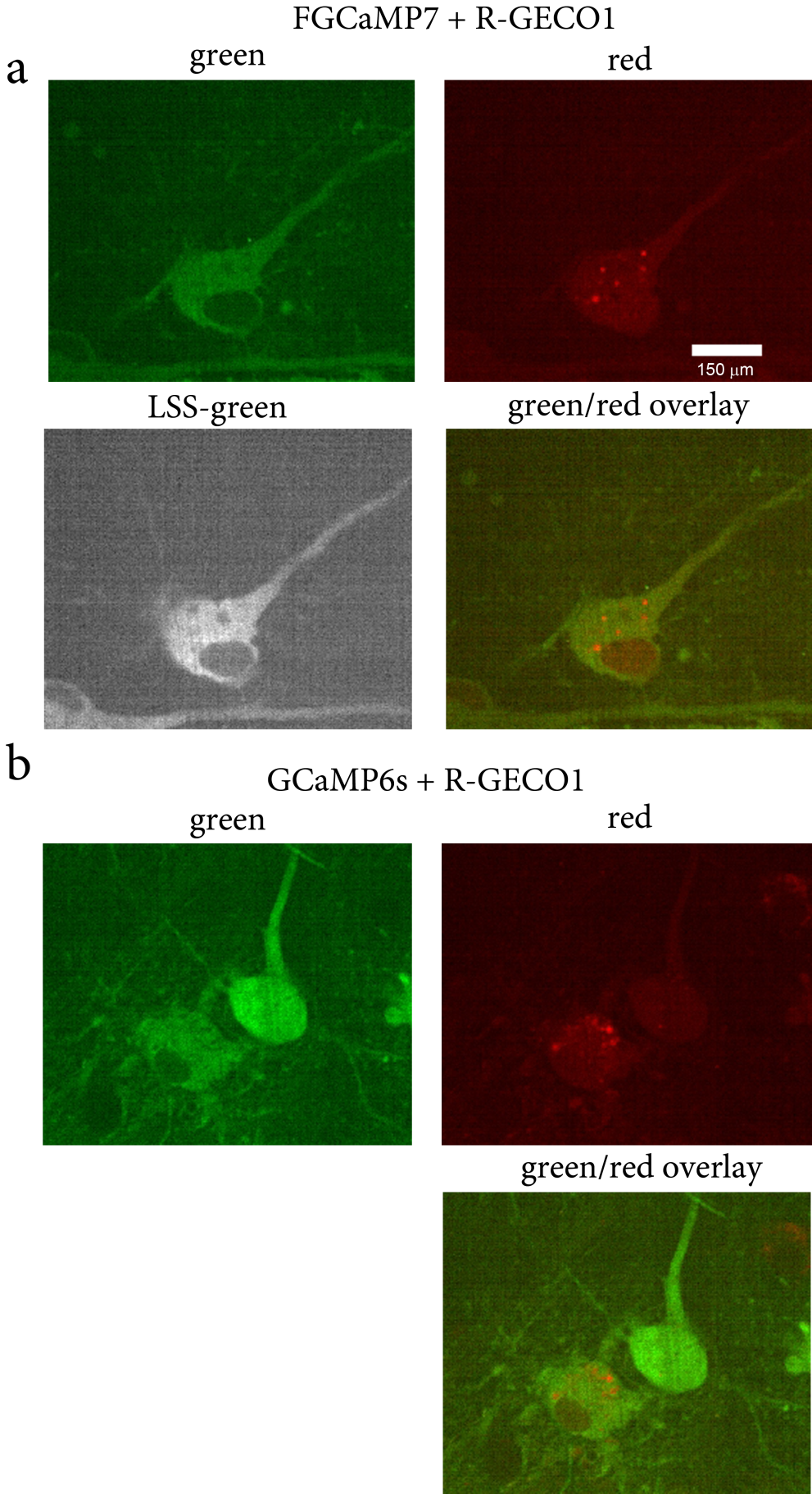


**Figure S11. Localization of FGCaMP7 (a) and GCaMP6s (b) GECIs co-expressed with R-GECO1 in the cultured neurons.** Confocal images of neurons co-expressing respective green indicator at 488 nm (green) and/or 405 nm (LSS-green) excitations with even distribution and red indicator R-GECO1 at 561 nm excitation (red) with uneven puncta-like distribution. Cultures were imaged on DIV 13 **(a)** and 16 **(b)**. Scale bar: 150 μm.


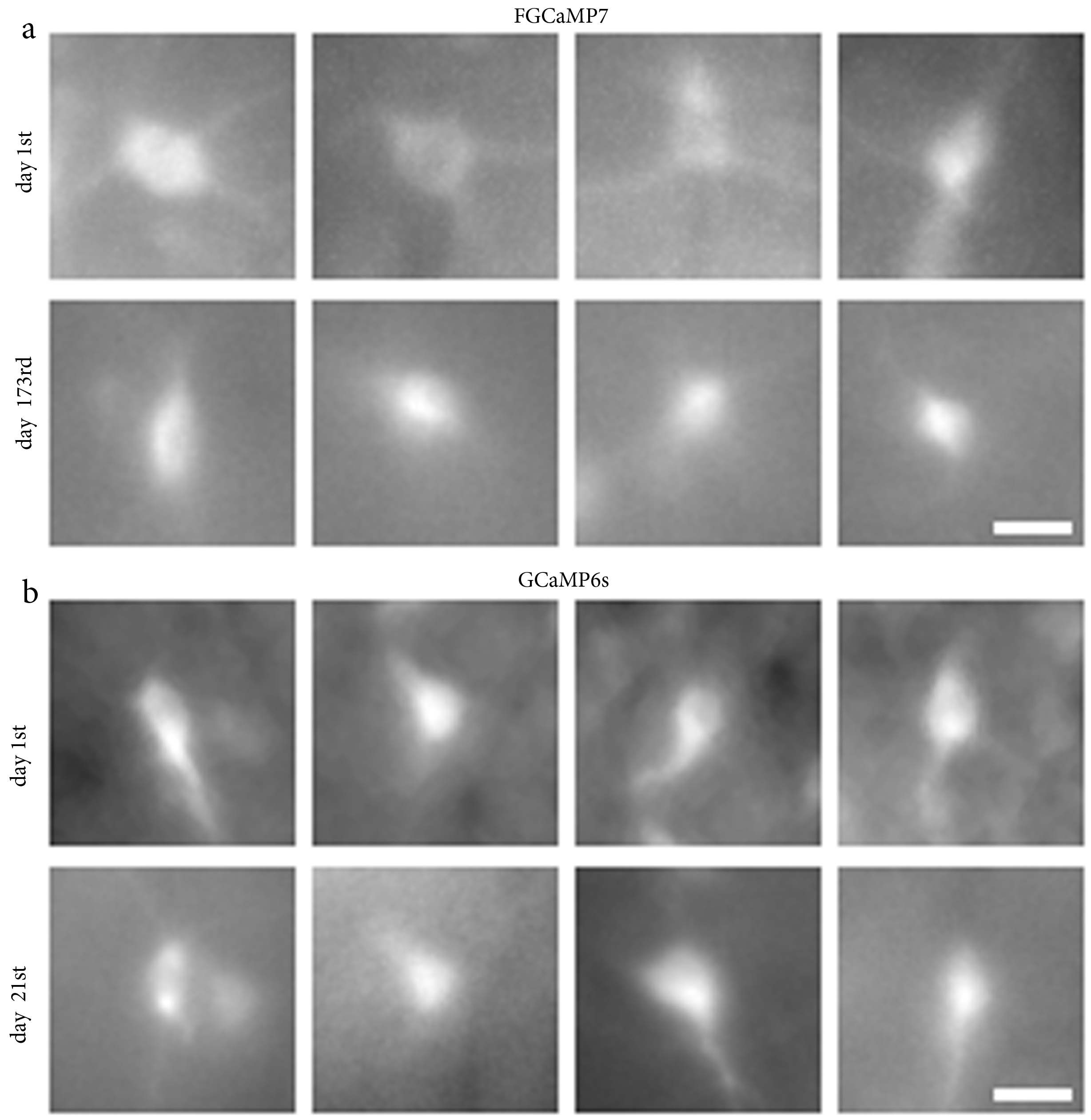


**Figure S12. Examples of localization of FGCaMP7 (a) and GCaMP6s (b) GECIs expressed in the hippocampus of mice.** Examples of individual cells expressing the FGCAMP7 (**a**) or GCaMP6s (**b**) indicators, which were imaged with the NVista HD miniscope at the start of the experiment (upper row) and 173 (a) or 21 (**b**) days later (bottom row, different cells within the same animal and field of view). Scale bar: 25 µm.


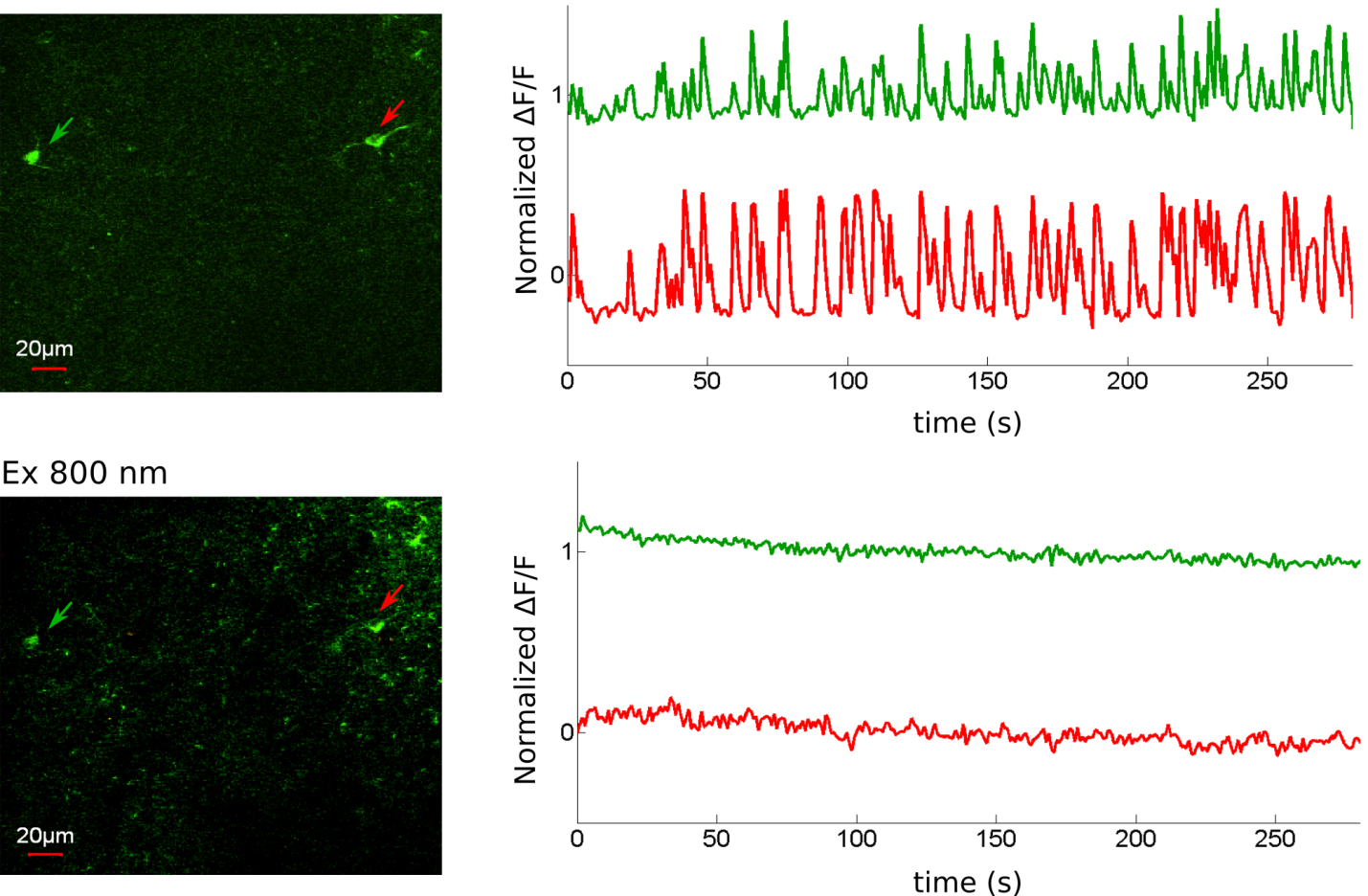


**Figure S13.** **In vivo neuronal Ca^2+^ activity in visual cortex of awake mice vizualized using two-photon microscopy. (left)** Two-photon images of V1 layer 2/3 neurons acquired during spontaneous activity at 960 nm and 800 nm excitation, respectively, in the mice expressing FGCaMP7 calcium indicator. **(right)** Sample traces obtained for neurons marked at images at 960 nm and 800 nm excitations. ΔF/F_0_ value normalized to maximal ΔF/F_0_ for each trace.

**
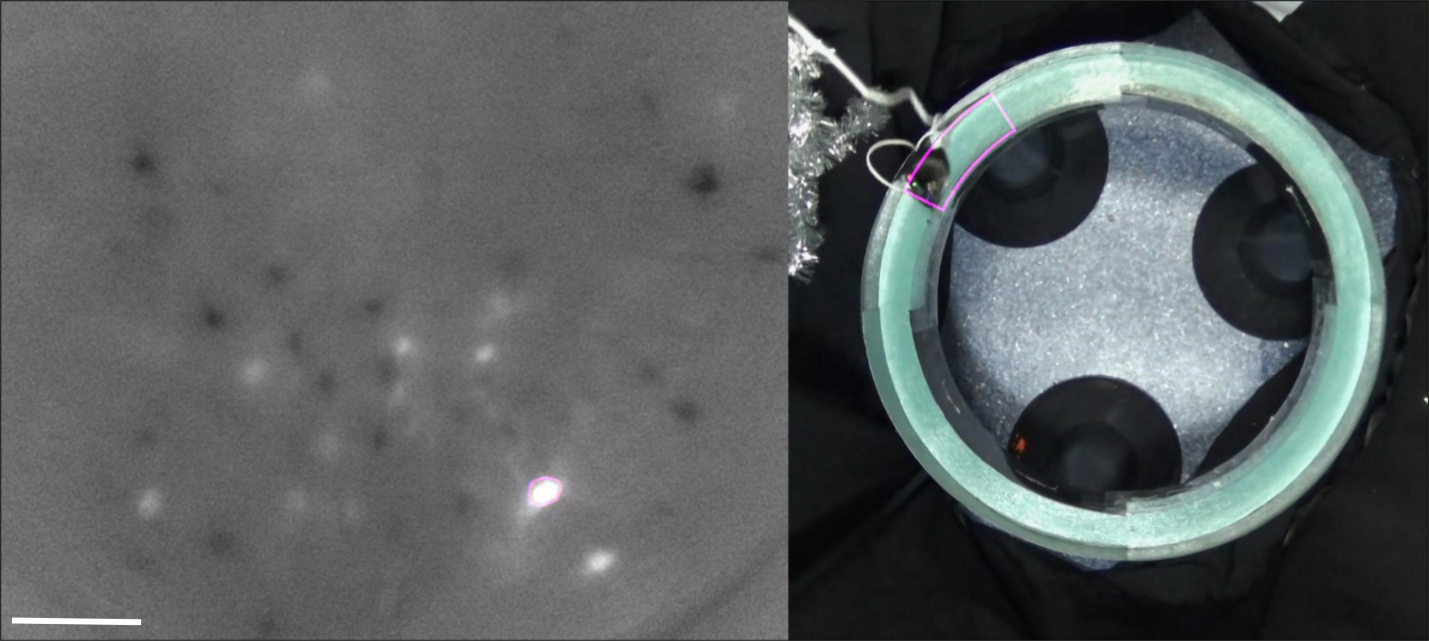
**

**Video S1. *In vivo* place cell activity in the CA1 subregion of hippocampus of mouse visualized with the FGCaMP7 calcium indicator and nVista HD miniscope synchronized with mouse position**. A video of place cell calcium activity in CA1 area of mouse hippocampus displayed as ΔF/F changes in fluorescence at 20 Hz frame rate (left) synchronized with mouse movement in O-shaped track (right). GLP 1040 lens probe was used. Video is shown for a time from 4.5 to 6.5 min. Scale bar: 100 µm. The identified place cell is marked by a circle and corresponds to the cell shown in Main text, Figure 6d.

**
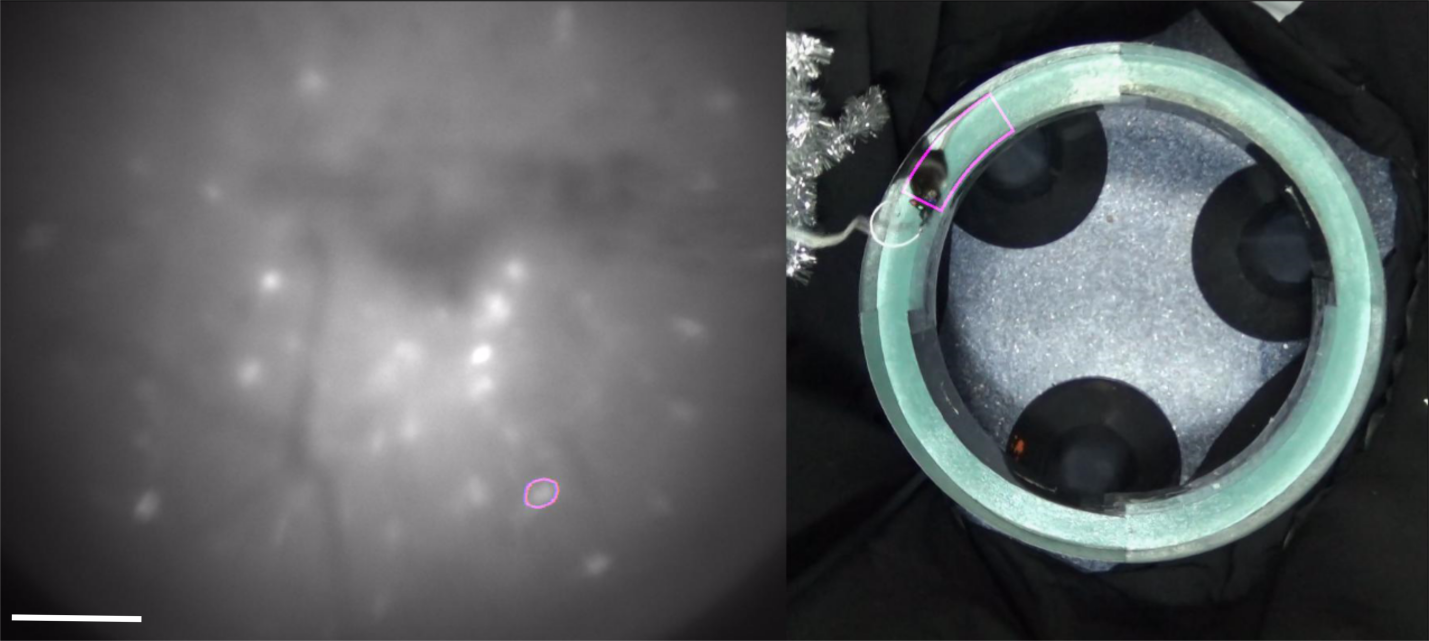
**

**Video S2. *In vivo* place cell activity in the CA1 subregion of hippocampus of mouse visualized with the FGCaMP7 calcium indicator and nVista HD miniscope synchronized with mouse position**. A video of place cell calcium activity in CA1 area of mouse hippocampus displayed in raw fluorescent units at 20 Hz frame rate (left) synchronized with mouse movement in O-shaped track (right). GLP 1040 lens probe was used. Video is shown for a time from 4.5 to 6.5 min. Scalebar: 100 µm. The identified place cell is marked by a circle and corresponds to the cell shown in Main text, Figure 6d.

**Supplementary references**

1. Tsien, R. Y., The green fluorescent protein. *Annu Rev Biochem* **1998,** 67, 509-44.

2. Subach, O. M.; Cranfill, P. J.; Davidson, M. W.; Verkhusha, V. V., An enhanced monomeric blue fluorescent protein with the high chemical stability of the chromophore. *PLoS One* **2011,** 6, (12), e28674.

3. Barykina, N. V.; Doronin, D. A.; Subach, O. M.; Sotskov, V. P.; Plusnin, V. V.; Ivleva, O. A.; Gruzdeva, A. M.; Kunitsyna, T. A.; Ivashkina, O. I.; Lazutkin, A. A.; Malyshev, A. Y.; Smirnov, I. V.; Varizhuk, A. M.; Pozmogova, G. E.; Piatkevich, K. D.; Anokhin, K. V.; Enikolopov, G.; Subach, F. V., NTnC-like genetically encoded calcium indicator with a positive and enhanced response and fast kinetics. *Sci Rep* **2018,** 8, (1), 15233.
